# Indiana State Dental Association

**Published:** 1868-05

**Authors:** John F. Johnston


### INDIANA STATE DENTAL ASSOCIATION.

THIS Society will meet in the city of Indianapolis, on the last Tuesday in June, 1868, at 2 P. M. Preparations for Demonstrations in the Operating Chair have been made, Essays written, and a session of more than ordinary interest is expected. The profession throughout the country are *cordially* invited, and those in our own State are earnestly urged to come up *en masse*.

JOHN F. JOHNSTON, *Secretary.*

